# The Relationships Between Plasma Adrenomedullin and Endothelin-1 Concentrations and Doppler Echocardiographic Indices of Left Ventricular Function During Static Exercise in Healthy Men

**DOI:** 10.2478/v10078-012-0047-z

**Published:** 2012-07-04

**Authors:** Krzysztof Krzemiński, Wiesława Pawłowska-Jenerowicz

**Affiliations:** 1 Department of Applied Physiology, Mossakowski Medical Research Centre, Polish Academy of Sciences, Warsaw, Poland.; 2 Outpatient Cardiac Unit for Diagnosis and Therapy, Mossakowski Medical Research Centre, Polish Academy of Sciences, Warsaw, Poland.

**Keywords:** Static handgrip, adrenomedullin, endothelin-1, catecholamines, hemodynamics, Doppler echocardiography

## Abstract

Our previous study showed a significant relationships between static exercise-induced changes in plasma adrenomedullin (ADM) and those in endothelin-1 (ET-1), noradrenaline (NA) and pre-ejection period/left ventricular ejection time ratio (PEP/LVET) in older healthy men. It is hypothesized that ADM, ET-1, NA and adrenaline (A) may function as endogenous regulators of cardiac function by modulating myocardial contractility during static exercise. The present study was undertaken to assess the relationships between exercise-induced changes in plasma ADM, ET-1, NA, A concentrations and those in ascending aortic blood flow peak velocity (PV) and mean acceleration (MA) measured by Doppler echocardiography in 24 healthy older men during two 3-min bouts of handgrip at 30% of maximal voluntary contraction, performed alternately with each hand without any break between the bouts. Plasma ADM, ET-1, NA and A as well as heart rate (HR), blood pressure (BP), PV and MA were determined. During handgrip, plasma ADM, ET-1, NA and A as well as HR, BP increased, whereas PV and MA decreased. The increases in plasma ADM correlated positively with those in ET-1, NA and diastolic BP, and correlated negatively with changes in PV (r= −0.68) and MA (r= −0.62). The increases in plasma ET-1 correlated positively with those in NA and BPs and correlated negatively with changes in PV (r= −0.67) and MA (r= −0.60). The results of this study suggest that in healthy older men the exercise-induced changes in plasma ADM, ET 1 and catecholamines are related to alterations in left ventricular contractile state and may co-operatively counteract age-related deterioration of cardiac performance in men.

## Introduction

The control mechanisms involved in cardiovascular and neurohormonal responses to static exercise are not fully understood. The available literature shows that some neurohormones are involved in the circulatory response to this exercise in both healthy men and patients with coronary artery disease, heart failure (HF) and hypertension. It was demonstrated that adrenomedullin (ADM) and endothelin-1 (ET-1) contribute to the alterations in systemic vascular resistance and that ADM may act to improve left ventricular function during static exercise, especially in patients with heart failure and healthy older men (Mangieri et al., 1997; Krzeminski et al., 2012). ADM (a vasodilator) and ET-1 (a vasoconstrictor) are peptide hormones produced and released mainly from endothelial cells. However, these peptides and their binding sites have also been identified in the heart (Tsuruda et al., 2001; Haynes et al., 1998). Hence, the peptides are thought to be synthesized and released within the myocardium and to act in an autocrine-paracrine fashion. The role of ADM and ET-1 in the modulation of myocardial contractility is not clear. Some studies suggest that these peptides exert a positive inotropic effect (Szokodi et al., 1998; Pan et al., 2007; De Giusti et al., 2008; Takanashi et al., 1991), whereas other show a negative inotropic effect (Ikenouchi et al., 1997), or no effect (Bäumer et al., 2002; Stangl et al., 2000). It seems likely that ET-1 and ADM with their opposing vasoactive properties and modulatory influences on myocardial inotropy play important roles in maintaining vascular tone and modulating myocardial contractility during static exercise.

Several research teams demonstrated a decline in left ventricular contractile function in response to static exercise as evidenced by a significant reductions in fractional shortening, mean velocity of circumferential fiber shortening, ascending aortic blood flow velocity and acceleration, or by an increased pre-ejection period to left ventricular ejection time ratio (PEP/LVET- an inverse index of left ventricular myocardial performance). These alterations were explained by failure of the heart to respond to acutely increased left ventricular afterload (Bamrah et al., 1991; Krzeminski et al., 2009, 2012).

Since our previous study performed in older healthy men (Krzeminski et al., 2012) showed correlations between handgrip-induced changes in plasma ADM and those in plasma ET-1, noradrenaline (NA), pre-ejection period (PEP) and PEP/LVET, it seemed of interest to extend these data to the Doppler echocardiographic measurements of ascending aortic blood flow velocity such as peak velocity and acceleration, which are considered more sensitive to changes in cardiac contractility than systolic time intervals. Our hypothesis is that both ADM and ET-1, with their positive inotropic properties might be involved in the compensatory mechanisms preventing deterioration of left ventricular systolic function during static effort in healthy older men. Thus, we determined cardiovascular indices including heart rate, blood pressure, peak velocity and mean acceleration of ascending aortic blood flow and the endocrine variables including plasma concentrations of ADM, ET-1, NA and adrenaline (A) during static handgrip exercise. This exercise was chosen as the stimulus since it evokes activation of the sympathetic nervous system and an acute marked increase in afterload, which affects myocardial contractility (Siegel et al., 1972).

## Material and Methods

### Subjects

The study was performed in 24 older (mean age 66.3 ±2.4 years) male volunteers. They were recruited from the general population by an advertisement and found to be in good health. All were normotensive, non-obese, non-smokers and were not taking any medication. A comprehensive clinical evaluation was performed in all subjects by physician, with testing including exercise electrocardiography, echocardiography, hematological and multipanel serum biochemistry screening. All the subjects gave their informed consent to participate in the study. The investigation conformed with the principles outlined in the Declaration of Helsinki and was approved by the Local Ethics Committee. General characteristics of the subjects is presented in Table 1.

#### Procedure

All the tests were carried out under similar environmental conditions (24°C and 40–50% relative humidity) between 4:00 and 5:00 P.M. Each subject had the maximal voluntary contraction (MVC) of the right and left hand determined using hand dynamometers (Medipan, Poland). Then, they had a catheter inserted into the antecubital vein in one arm and were allowed to rest in the supine position for 30 min. After the rest period, blood samples were taken for determinations of baseline plasma adrenomedullin, noradrenaline, adrenaline and endothelin-1 concentrations. Next, the subjects performed 3-min handgrip at 30% MVC with right hand and then 3-min handgrip at the same percentage of MVC with left hand, with no resting interval between the bouts, and more blood samples were taken at the end of each 3-min exercise bout, and 5-min after termination of the exercise. To avoid Valsalva manoeuvre, the subjects were instructed not to hold their breath during the handgrip bouts. The subjects respiratory pattern was monitored continuously during the experiment. The protocol with two exercise bouts was used with the intention to prolong the duration of the stimulus, since the static handgrip at 30% MVC performed by one hand cannot usually be maintained longer than 3–4 min, which was thought to be too short time period for marked activation of the endocrine system.

### Measurements

#### Biochemical analysis

All plasma hormone determinations were performed in duplicate. The plasma ADM was determined using a specific and sensitive radioimmunoassay kit for ADM (1–52) produced by Phoenix Pharmaceuticals Inc., Belmont 94002 CA, USA. The limit of detection for this assay was 0.5 pg ADM per tube, and the half-maximal inhibition dose of radiodinated ligand binding was 10 pg ADM per tube. The intra-assay coefficient of variance was 5.8%. Plasma concentrations of catecholamines were measured by radioimmunoassay using 2 CAT RIA tests produced by Bio Source Europe S.A., Nivelle, Belgium. The sensitivity of the assay was 37.5 pg/ml and 7.5 pg/ml for NA and A, respectively; whereas the intra-assay coefficients of variance were 4.7% and 4.9%, respectively. Plasma ET-1 was assessed using commercial ELISA kit BI-20052 Endothelin (Biomedica, Austria). The sensitivity of the assay was 0.1 fmol/ml, and the intra-assay coefficient of variation was 3.9%.

#### Echocardiographic examination

Doppler echocardiographic recordings of ascending aortic blood flow velocities were obtained in the continuous-wave mode with a 2.0 MHz non-imaging Doppler transducer (Vivid 5 Gems Ultrasound, Tirat Carmel, Israel) held in the suprasternal notch. The transducer was angulated anteriorly and to the right to obtain the best Doppler signal, as judged by the sharpness of outline of the complexes on the visual display and by the pitch of the audio sound of the Doppler shift frequencies. During the exercise, no change in the position and tilt of the transducer and no modification in the setting of control switches were permitted. All Doppler signals with an ECG (lead II) and a phonocardiogram superimposed were recorded on a strip-chart recorder at a paper speed of 100 mm/s. Spectral envelopes of minimum six consecutive loops with the highest peak velocities were analyzed at rest, during the third and last minute of exercise and 5-min after termination of the test. Peak velocity (PV) of blood flow in the ascending aorta and time to peak velocity (TP, the time from the onset of flow to time at which velocity of flow was maximal) were measured. Acceleration of blood flow in the ascending aorta was calculated as PV/TP and averaged over all measurements for a given patient to yield MA. Off-line measurements were performed by two independent investigators and the data were averaged. Preliminary studies of 24 recordings obtained in six subjects at rest and during static exercise revealed intraobserver variability of less than 9%, that is no more than 0,1 m/s in any recording.

Left ventricular ejection fraction was determined at rest, just before exercise, using the 2-D method of Simpson. Heart rate was determined from the R-R interval of the ECG. Blood pressure was measured just before, at the third and at the last minute of exercise, and 5-min after termination of the test by auscultation on the non-exercising arm.

### Statistics

The data are presented as the mean ±SEM. Differences between the pre-exercise and exercise values of the measured variables were evaluated with the Student t-test for paired data. P<0.05 was considered significant. Correlations coefficients were calculated between variables using the linear regression analysis. All the statistical analyses were performed using the Statistica v. 6 statistical software package (Statsoft Inc., Tulsa, OK., USA).

## Results

### Adrenomedullin, endothelin-1, noradrenaline, and adrenaline

Handgrip significantly elevated plasma ADM, NA and A already in the 3rd min of exercise (p<0.001) and plasma ET-1 at the end of exercise (p<0.01) in all subjects. The values of ADM, NA, and A obtained at the 6th minute of exercise were significantly higher than those at the 3rd minute (p<0.001). At the 5th min of the recovery period, plasma ADM was significantly higher than that before exercise whereas plasma NA, A and ET-1 concentrations did not differ significantly from the resting values (Fig. 2).

Significant positive relationships were ascertained between baseline values of plasma ADM and NA concentrations (r= 0.650, p<0.001), and between the exercise-induced increases in plasma ADM (expressed as percentage of baseline values) and those in NA and ET-1 concentrations (r= 0.710, p<0.001; r= 0.680, p<0.001; respectively). The exercise-evoked increases in plasma ET-1 concentrations (expressed as percentage of baseline values) correlated positively with those in plasma NA (r= 0.598, p<0.001).

### Heart rate, and blood pressure

**Figure 1:**
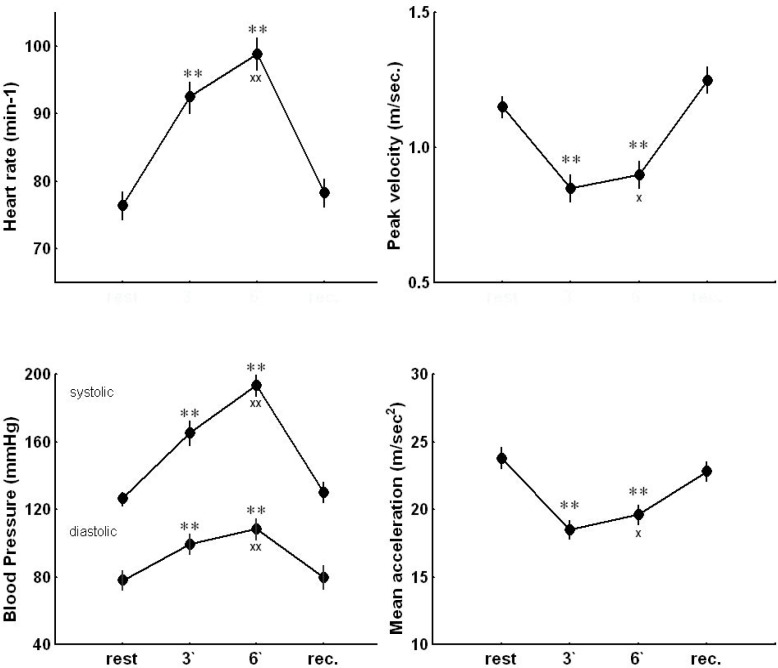
Heart rate, systolic and diastolic blood pressure, peak velocity and mean acceleration of blood flow in the ascending aorta at rest, during handgrip (3′ and 6′) and at the 5^th^ min of the recovery period (rec.). Values are means ± SEM. * p<0.05, ** p<0.01 versus resting value; ^x^ p<0.05, ^xx^ p<0.01 versus the value for the 3^rd^ minute of exercise.

**Figure 2:**
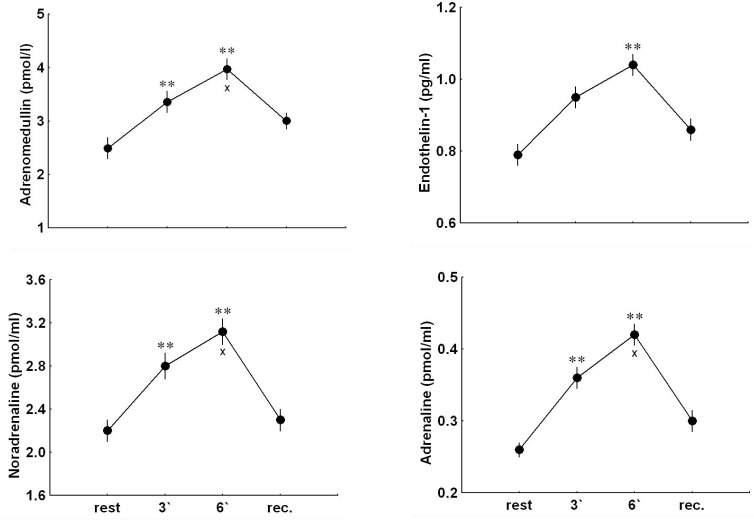
The plasma concentrations of adrenomedullin, noradrenaline, adrenaline and endothelin-1 at rest, during handgrip (3′ and 6′) and at the 5^th^min of the recovery period (rec). Values are means ± SEM; * p<0.05, ** p<0.01 versus resting value;^x^p<0.05,^xx^p<0.01 versus the value for the 3^rd^minute of exercise.

The resting values of heart rate (HR), systolic (BPs) and diastolic (BPd) arterial blood pressures were within normal limits. The handgrip caused significant increases in HR, BPs and BPd (p<0.001) already at the 3rd min of exercise in all subjects. The values obtained at the 6th min were significantly higher than those at the 3rd minute of exercise (p<0.001). After 5 min recovery period, HR, BPs and BPd returned to the resting values (Fig. 1).

Significant positive correlations were ascertained between the exercise-induced increases in BPs (expressed as percentage of baseline values) and those in plasma ET-1 (r= 0.697, p<0.001) as well as between the exercise-induced increases in BPd and those in plasma ADM (r= 0.789, p<0.001).

### Doppler echocardiographic indices of left ventricular systolic function

The resting values of PV and MA were within normal limits. The static handgrip caused declines in PV (p<0.001) and MA (p<0.01) in all subjects. The decreases in PV and MA during the second bout of exercise were significantly lower than those during the first bout (p<0.05). After 5 min recovery period, PV and MA did not differ significantly from the resting values (Fig. 1).

**Figure 3:**
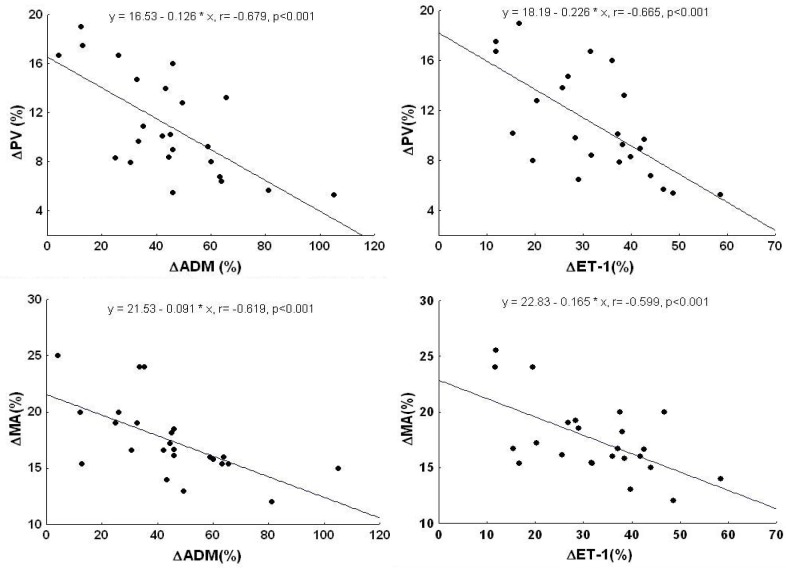
*Correlations between the exercise-induced changes in plasma adrenomedullin (ΔADM) and those in peak velocity (ΔPV) and mean acceleration (ΔMA) as well as between the exercise-induced changes in plasma endothelin-1 (ΔET-1) and those in ΔPV and ΔMA. All variables are expressed as percentage of baseline values*.

Significant relationships were found between the exercise-induced decreases in both PV and MA (expressed as percentage of baseline values) and increases in plasma ADM (r=−0.679, p<0.001 and r=−0.619, p<0.001; respectively) and ET-1 (r=−0.665, p<0.001 and r=−0.599, p<0.001; respectively; Fig. 3).

The exercise-induced changes in PV and MA (expressed as percentage of baseline values) significantly correlated also with those in both plasma NA and A (r=−0.624, p<0.001; r=−0.598, p<0.001 and r=−0.610, p<0.001; r=−0.586, p<0.001; respectively).

## Discussion

It has been generally accepted that Doppler echocardiographic measurements of ascending aortic blood flow velocity are sensitive enough to detect changes in left ventricular systolic performance (Wallmeyer et al., 1988). The peak velocity and mean acceleration of ascending aortic blood flow correlate well with the invasively measured maximal rate of pressure rise during ventricular contraction (peak dP/dt – the ratio of the rise in pressure during isovolumetric contraction to the isovolumetric contraction time). The peak dP/dt is sensitive to changes in myocardial contractility, insensitive to changes in afterload, and only mildly affected by changes in preload (Rhodes et al., 1993).

The present data show that, in older healthy men, a moderate intensity static handgrip results in declined peak velocity and mean acceleration of ascending aorta blood flow (as measured by Doppler echocardiography) and increased plasma concentrations of ADM, ET-1 and catecholamines. Furthermore, exercise-induced changes in plasma ADM, ET-1, NA and A correlate negatively with those in PV and MA.

The static exercise-related alterations in PV and MA are consistent with the findings of Bamrah et al. (1991) who reported significant declines of peak velocities and mean acceleration with static handgrip in healthy subjects and patients with coronary artery disease or heart failure. Those authors concluded that an acute increase in afterload with static exercise is not sufficiently counteracted by a moderately augmented contractile state and results in reduction of peak velocity of ascending aorta blood flow.

Several studies demonstrated a significant reduction in fractional shortening and mean velocity of circumferential fiber shortening as well as an increase in end-systolic diameter during static exercise in cardiac patients (Heath et al., 1982; Grossman et al., 1973; Siegel et al., 1972). The factors responsible for impaired left ventricular function during static exercise include an increase in left ventricular wall tension and myocardial ischemia due to the exercise-induced coronary vasoconstriction (Øie et al., 2000; Aung-Din et al., 1981). The latter changes cause release of endogenous regulators of cardiac function, including catecholamines, ADM, ET-1, angiotensin II and natriuretic peptides, from various types of cells. It seems that cardiac contraction may be regulated by cross-talk among the peptide hormones. It has been shown that the ADM and ET-1 can be synthesized and secreted from human cardiac myocytes and that the expression and function of ADM receptors is modulated by humoral and mechanical factors in myocardium (Hirayama et al., 1999). Mishima et al. (2001) have found that ET-1 stimulates ADM production and release from cultured rat cardiomyocytes as well as up-regulates ADM receptors such as calcitonin receptor like receptor (CRLR) and receptor activity modifying protein (RAMP-3), thereby enhancing the intracellular cAMP response to ADM. Adrenomedullin has been reported to activate adenylate cyclase-cAMP system in isolated cardiac myocytes, which is one of the major pathways for the regulation of myocardial contractility (Sato et al., 1997). However, Szokodi et. al. (1998) found that cAMP is not the major second messenger of the inotropic action of ADM, and suggested that ADM-induced inotropic positive action may involve both Ca2+ release from ryanodine- and thapsigargin-sensitive intracellular Ca2+ pools and an enhanced Ca2+ influx from sarcoplasmic reticulum through L-type Ca2+ channels. Some authors suggested, that ADM acts mainly as a regulator of vascular tone and thereby influence myocardial perfusion. As a secondary effect, contractility may be affected (Bäumer et al., 2002). Additional finding of the present investigation is that the exercise-induced increases in plasma ADM concentrations correlated positively with those of BPd. This is in agreement with our previous data and indicate that some hemodynamic changes stimulate ADM secretion (Krzeminski et al., 2009).

In our study, exercise-induced changes in plasma ADM correlated positively with those in plasma ET-1 and NA, and there was a positive correlation between changes in plasma ET-1 and NA. Rat studies showed that NA increases ADM production and secretion from endothelial cells by stimulation of protein kinase C through the alpha-1 receptors and cAMP through beta receptors (Isumi et al., 1998). In a study conducted by Roessler et al. (2011) a marked increase in plasma ADM concentration was observed after A infusion during head up tilt in humans. However, we found no significant correlation between plasma A and ADM levels.

It is well established that ET-1 increases the plasma NA concentration, whereas NA facilitates the expression of prepro-ET-1 mRNA and the production of ET-1. Chu et al. (2003) found that the myocardial contractility in the dog is regulated by crosstalk between NA and ET-1 through different signaling pathways whose activation depends on the concentration of NA. Endothelin-1 has a positive inotropic effect in the presence of NA at low concentrations (0.1–1.0 nM), negative at high NA concentrations (around 100 nM) and a biphasic facilitatory/inhibitory effects at intermediate levels. The positive inotropic effect of ET-1 in the presence of NA requires the simultaneous activation of protein kinase A (PKA) and protein kinase C (PKC) signaling pathways, and the activation of Gs protein-coupled cAMP/PKA pathway or G protein-coupled cGMP/PKG pathway (Zerkowski et al., 1993). However, it may also involves an activation of the Na+/H+ exchanger, increase in Ca2+ transient or activation of an intracellular pathway triggered by mitochondrial reactive oxygen species formation (Krämer et al., 1991; De Giusti et al., 2008; Chu et al., 2005).

Mac Carthy et al. (2000) demonstrated that selective ETA receptor antagonist markedly increased myocardial contractility in cardiac patients with reduced ejection fraction and decreased it in healthy men with normal ejection fraction. The authors concluded that effects of ET-1 on myocardial contractility were mediated through the ETA receptor and that ET-1 may have a supporting role on cardiac function in healthy subjects.

In summary, the results of this study suggest that ADM, ET-1, NA and A, due to their inotropic positive properties, may co-operatively counteract age-related deterioration of cardiac performance in men.

## Figures and Tables

**Table 1:** Characteristics of the subjects (the values are means ± SEM, n=24)

Age [yrs]	Height [cm]	Body mass [kg]	BMI [kg/m^2^]	Ejection fraction [%]
66.3 ±2.4	178.1 ±3.5	83,1 ±5.2	26.1 ±0.6	50,3 ±1.43

BMI – body mass index
